# Organotypic Rat Testicular Organoids for the Study of Testicular Maturation and Toxicology

**DOI:** 10.3389/fendo.2022.892342

**Published:** 2022-06-09

**Authors:** Sadman Sakib, Nathalia de Lima e Martins Lara, Brandon Christopher Huynh, Ina Dobrinski

**Affiliations:** ^1^ Department of Biochemistry and Molecular Biology, Cumming School of Medicine, University of Calgary, Calgary, Canada; ^2^ Department of Comparative Biology and Experimental Medicine, Faculty of Veterinary Medicine, University of Calgary, Calgary, Canada; ^3^ Department of Biological Sciences, Faculty of Science, University of Calgary, Calgary, Canada

**Keywords:** testicular organoid, spermatogonia, MEHP, cadmium chloride, Sertoli cell

## Abstract

An *in vitro* system to study testicular maturation in rats, an important model organism for reproductive toxicity, could serve as a platform for high-throughput drug and toxicity screening in a tissue specific context. *In vitro* maturation of somatic cells and spermatogonia in organ culture systems has been reported. However, this has been a challenge for organoids derived from dissociated testicular cells. Here, we report generation and maintenance of rat testicular organoids in microwell culture for 28 days. We find that rat organoids can be maintained *in vitro* only at lower than ambient O_2_ tension of 15% and organoids cultured at 34°C have higher somatic cell maturation and spermatogonial differentiation potential compared to cultures in 37°C. Upon exposure to known toxicants, phthalic acid mono-2-ethylhexyl ester and cadmium chloride, the organoids displayed loss of tight-junction protein Claudin 11 and altered transcription levels of somatic cell markers that are consistent with previous reports in animal models. Therefore, the microwell-derived rat testicular organoids described here can serve as a novel platform for the study of testicular cell maturation and reproductive toxicity *in vitro*.

## Introduction

Male factor infertility is responsible for 40-50% of all cases of infertility worldwide (1) and around 9-16% of all men suffer from infertility (2). The causes of infertility are varied; some are due to pathophysiological conditions, while others may be caused by environmental toxicants such as exposure to phthalates and heavy metals (3–5).

Spermatogenesis is a highly orchestrated process that is dependent on a tightly regulated stem cell niche (6). This spermatogonial stem cell niche is primarily composed of spermatogonia and somatic cells, such as Sertoli cells, peritubular myoid cells and Leydig cells (6). The regulation of spermatogonial cell fate is tightly modulated by these somatic cells (7, 8). *In vitro* culture systems to model this niche have primarily been limited to conventional two-dimensional co-culture systems (9), which fail to mimic the cell-cell signaling seen *in vivo* (10, 11). A three-dimensional organotypic culture system can bridge the gap between cell cultures and whole animal models to better study developmental processes such as spermatogonial differentiation and reproductive toxicology *in vitro* (12, 13). Several three-dimensional testicular organoid systems have been developed with this goal in mind (11, 14–18).

Here, we describe the adaptation of microwell-derived testicular organoids, previously established in porcine, murine, human and primate models (11), to rat testicular organoids and highlight the species-specific challenges encountered during this endeavor. Rats have been extensively studied as an animal model for testicular maturation and reproductive toxicology (19–22). We report here that organotypic rat testicular organoids can be derived and maintained for 28 days only at lower than ambient (15%) O_2_ tension. We also characterize rat organoids cultured at both 37°C and 34°C and show that rat organoids cultured at lower temperature better support the differentiation of spermatogonia. Finally, we present proof of principle with known environmental toxicants to establish the utility of rat testicular organoids for the study of reproductive toxicity.

## Materials and Methods

### Preparation of Rat Starting Testicular Cell Population

SAS Sprague Dawley rats (Strain: code 400, Charles River), aged P4-P5, were euthanized and testes were removed. All animal procedures were performed as approved by the Animal Care Committee, University of Calgary. Using a pair of forceps, the tunica albuginea was removed to release the tubules, which were then washed in Hank’s Balanced Salt Solution (HBSS, Gibco, cat# 14025092) containing 1% penicillin-streptomycin (ThermoFisher Scientific, cat# 15070063). The tubules were digested using collagenase type IV (Worthington-Biochem, cat# LS004189) in HBSS (2 mg/mL) for 25 min at 37°C. The tubules were then sedimented by centrifugation at 90x g for 1.5 min and washed with 5 mL HBSS thrice. Finally, the tissue was digested with 0.25% trypsin–EDTA (Sigma-Aldrich, cat# T4049) and DNase I (Sigma-Aldrich, cat# DN25) in HBSS (10 mg/mL) for 5 min to obtain the starting cell population (11). All experiments were replicated using a minimum of three independently prepared cell suspensions.

### Generation of Organoids

AggreWell 400 plates (STEMCELL Technologies Inc, Vancouver, Canada, cat# 34450) were treated with Anti-adherence Rinsing Solution (STEMCELL Technologies Inc, Vancouver, Canada, cat# 07010) according to the manufacturer’s instructions. Each well was filled with 500 μL of organoid formation medium (OFM) (Dulbecco Modified Eagle Medium F/12 (Gibco, cat# 11330-032) supplemented with insulin 10 μg/mL, transferrin 5.5 μg/mL, selenium 6.7 ng/mL (Gibco, cat#41400-045); 20 ng/mL epidermal growth factor (R & D Systems, cat# 236-EG); 1% Penicillin-Streptomycin) and the plates were then centrifuged at 2000x g for 2 minutes to release any trapped air bubbles (11). Each well was then seeded with 6 × 10^5^ rat testicular cells suspended in 500 μL OFM. Finally, the plates were centrifuged at 500x g for 5 minutes to sediment the cells in the microwells. 500 μL of media were removed from each well and fresh media supplemented with Corning Matrigel Growth Factor Reduced (GFR) Basement Membrane Matrix (1:100 dilution; Life Sciences, cat# 354230) was used to replenish each well. Microwell plates were divided into three groups and placed in 3 incubators setup for three conditions: (i) ambient O_2_ tension (18.4% or 6.34 mM O_2_, 5% CO_2_, 37°C), (ii) 15% O_2_ tension at 37°C (15% or 5.18 mM O_2_, 5% CO_2_) and (iii) 15% O_2_ tension at 34°C (15% or 5.22 mM O_2_, 5% CO_2_). Culture in OFM was carried out for 3-5 days with 50% media changes every second day.

### Maturation and Differentiation of Organoids

After culturing the organoids for 3 days, which were designated as day 0 undifferentiated organoids, the OFM media was completely removed, and the culture was continued in organoid differentiation medium (ODM). ODM was composed of Minimum Essential Medium α (ThermoFisher Scientific, cat# 12571063) supplemented with 10% KnockOut Serum Replacement (ThermoFisher Scientific, cat# 10828028), hepatocyte growth factor (5 ng/mL) (R&D Systems, cat# 294-HG), activin A (100 ng/mL) (Sigma-Aldrich, cat# a4941), follicle stimulating hormone (1 ng/mL) (Sigma-Aldrich, cat# F4021), luteinizing hormone (1 ng/mL) (Sigma-Aldrich, cat# L5259), testosterone (1 μM) (Steraloids, cat# A6950-000), recombinant human BMP-4 (20 ng/mL) (R&D Systems, cat# 314-BP), recombinant human BMP-7 (20 ng/mL) (R&D Systems, cat# 354-BP), 3,3’,5-triodo-L-thyronine sodium (2 ng/mL) (Sigma-Aldrich, cat# T6397), l-ascorbic acid-2-glucoside (1 mM) (Matrix Scientific, cat# 092375) and 1% Penicillin-Streptomycin (22, 23). Culture was carried out for an additional 28 days, with full media changes every second day. The organoids were sampled every 7 days, including day 0, for analysis.

### Immunohistochemistry

Testes tissue from 43-day old rats were fixed in 4% paraformaldehyde, dehydrated with a gradient series of ethanol, and embedded in paraffin wax to prepare sections of 5 µm thickness. The rat starting cell populations and organoids were fixed using 2% paraformaldehyde and spun down on slides using cytospin centrifugation (1000 rpm for cells and 500 rpm for organoids) (Cytospin 4, Thermo Scientific). The samples were then permeabilized using a gradient series of methanol (24) and blocked with 10% donkey serum. The testes tissue was incubated with anti-γ-H2AX (Gamma H2A Histone Family X) (25) and anti-SYCP3 (Synaptonemal Complex Protein 3**)** (26) (**Supplementary Table 1**). For rat testicular cells, the slides were incubated with anti-GATA4 (GATA Binding Protein 4) (27), anti-VASA (DEAD-Box Helicase 4) (28), anti-α-SMA(alpha-Smooth Muscle Actin) (29), anti-3β-HSD (3 Beta-Hydroxysteroid Dehydrogenase) (28) (**Supplementary Table 1**). In addition to the antibodies mentioned above, rat organoids were also incubated with anti-Collagen IV (30), anti-Laminin (31), anti-Fibronectin (32), anti-Claudin 11 (33), anti-UCHL1 (Ubiquitin *C*-terminal Hydrolase L1), anti-TNP1 (Transition Protein 1) (34), anti-PRM1 (Protamine 1) (35), anti-ACR (Acrosin) (36) and anti-AR (Androgen Receptor) (37) antibodies overnight at 4°C (**Supplementary Table 1**). Fluorescence labelling was done with secondary antibodies conjugated with Alexa Fluor 488 and 555 (**Supplementary Table 1**). DAPI (4′,6-diamidino-2-phenylindole) (Vector, cat# H1200) was used for labelling the nuclei. The cells were analyzed using Zeiss Imager.M2 fluorescence microscopy and the percentages of testicular cell types were determined by counting the cells with ImageJ software. The organoids were analyzed using a Leica TCS-SP8 confocal laser scanning microscope with the Leica Las X software.

### Cell Number Quantification Within Organoids

Immunohistochemistry for VASA was performed on day 7 and 28 organoids, while SYCP3 staining was performed on day 28 organoids. Using confocal microscopy, organoids were selected blindly based on only DAPI and scanned across the z-axis to quantify the number of VASA^+ve^ and SYCP3^+ve^ cells in each organoid. From each of the three independent experiments (n = 3) performed, a total of 30 organoids were analyzed for VASA^+ve^ and 10 organoids were analyzed for SYCP3^+ve^ cell counts.

### Reverse Transcription Quantitative Polymerase Chain Reaction

RNA was isolated from 1200 organoids using RNeasy Micro Kit (QIAGEN, cat # 74004) and then reverse transcription was performed using SuperScript™ IV VILO™ Master Mix (Thermo Fisher Scientific, cat# 11756050). RT-qPCR with the primers listed in **Supplementary Table 2** was performed with a 7.500 Fast Real-Time PCR System (Applied Biosystems) using SsoFast Eva Green Supermix with Low ROX (Bio-Rad laboratories, cat# 1725211). The expression levels were presented relative to *Gapdh*. Statistical analysis was performed on the mean of ΔΔCt.

### MEHP and CdCl_2_ Treatment

Day 19 organoids were treated with 1 μM MEHP and 0.25 μM CdCl_2_. Controls were treated with equivalent volumes of DMSO. After 48 hours of treatment, at day 21, the organoids were harvested and analyzed with immunofluorescence and RT-qPCR.

### Dose Determination

Day 5 organoids were treated with 0.5, 1 and 1.5 μM of MEHP (Sigma, Cat# 796832) and 0.01, 0.05, 0.25 and 1.25 μM of CdCl_2_ (Sigma, Cat# 202908). The control groups for MEHP and CdCl_2_ were treated with equivalent volumes of DMSO for 1.5 μM of MEHP and 1.25 μM of CdCl_2,_ respectively. Controls of 1.5 μM and 1.25 μM DMSO were treated with equivalent volumes of phosphate buffered saline (Thermo Fisher Scientific, cat# 14190144). At day 7 (48 hours after treatment), the organoids were harvested and approximately 60 organoids suspended in 50 μL ODM were seeded in each well of a 96-well plate as duplicates to perform the MTT assay (Abcam, cat#** **ab211091) according to manufacturer’s instructions. Absorbance at OD 590 nm was measured using SpectraMax i3x plate reader (Molecular Devices). MTT assay was used to measure cellular metabolic activity as an indicator of cell viability.

### Statistical Analysis

All the results described here are from at least three independent experiments performed with three separately prepared rat starting cell population (n = 3). Data were analyzed using the GraphPad Prism 8 software. Unpaired two-tailed *t*-tests were performed for single comparisons between two groups. For more than two groups, one-way ANOVA with Tukey’s multiple comparison tests were performed. A value of p < 0.05 was set as the limit of statistical significance.

## Results

### Rat Testicular Cells Generate and Maintain Organotypic Testicular Organoids at 15% O_2_ Tension

**Figure 1:**
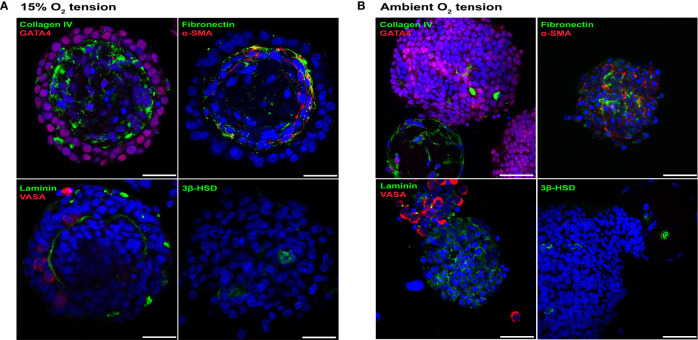
Rat testicular organoids cultured at 15% O_2_ tension maintained organotypic morphology. **(A, B)** Immunofluorescence images of day 6 rat testicular organoids at 15% **(A)** and ambient **(B)** O_2_ tensions showing the distribution of basement membrane (collagen IV, fibronectin, laminin), Sertoli cells (GATA4), peritubular myoid cells (α-SMA), germ cells (VASA) and Leydig cells (3β-HSD). Scale bars measure 25 μm.

A rat (P4-P5) testicular starting cell population which contained 92.2 ± 1.28% GATA4^+ve^ Sertoli cells (38), 1.7 ± 0.3% VASA^+ve^ germ cells (a marker for spermatogonia, spermatocytes and round spermatids) (39), 2.6 ± 0.7% 3β-HSD^+ve^ Leydig cells (40) and 19.97 ± 2.8% α-SMA^+ve^ peritubular myoid cells (41) (n = 3) was used to generate rat testicular organoids with organoid formation media (OFM). Since lower O_2_ tension is known to support higher differentiation potential of rat testicular cells (22), initial cultures were carried out in incubators set up for ambient and 15% O_2_ tensions (37°C). Both culture conditions supported the initial generation of testicular organoids (72 hours), with organotypic morphology similar to our previously published porcine and murine model (11). However, unlike the porcine or murine models (11), the rat organoids cultured in ambient O_2_ tension underwent a loss of testis-specific tissue architecture at day 6 of culture while day 6 organoids cultured at 15% O_2_ tension had distinct internal-interstitial and external-seminiferous epithelial compartments. The two compartments were separated by a collagen IV^+ve^, fibronectin^+ve^ and laminin^+ve^ basement membrane (**Figure 1A**). The external compartment was composed of VASA^+ve^ germ cells and GATA4^+ve^ Sertoli cells. α-SMA^+ve^ peritubular myoid cells were located lining the basement membrane in the interior compartment, while 3β-HSD^+ve^ Leydig cells were distributed throughout the interior compartment (**Figure 1A**). In contrast, the organoids cultured at ambient O_2_ tension showed increased Sertoli cell numbers, generation of large Sertoli cell clusters and complete or partial separation of internal and external compartments (**Figure 1B**). Thus, subsequent experiments were carried out at 15% O_2_ tension.

### Under Optimized Conditions, Rat Testicular Organoids Undergo Maturation and Support Spermatogonial Differentiation

**Figure 2:**
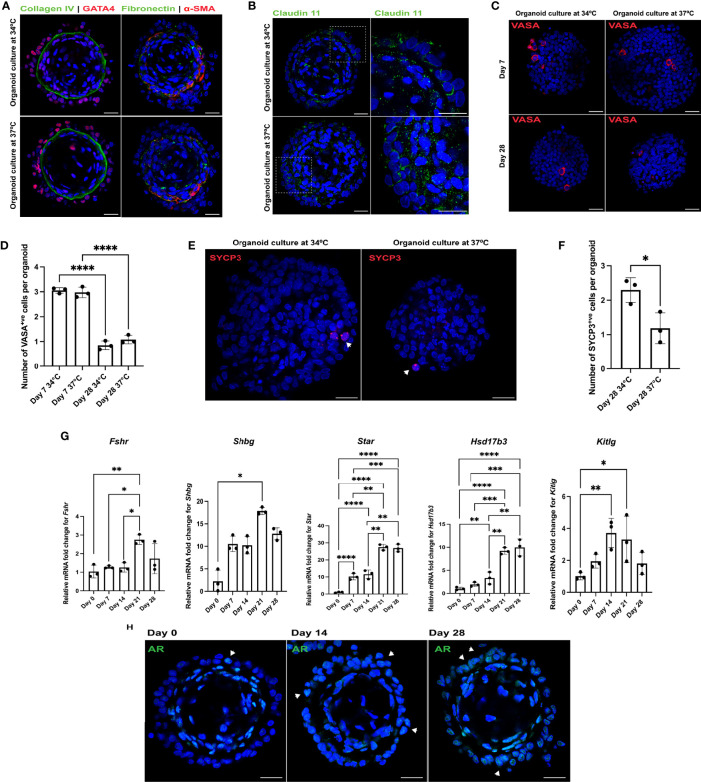
Rat testicular organoids support somatic cell maturation and spermatogonial differentiation at 34°C culture. **(A–C, E)** Immunofluorescent images of day 28 rat testicular organoids cultured at 34°C and 37°C showing **(A)** basement membrane (collagen IV, fibronectin), Sertoli cells (GATA4), peritubular myoid cells (α-SMA), **(B)** tight junction protein (Claudin 11) (inserts showing the magnified area on the right panel), **(C)** germ cells (VASA) and **(E)** meiotic cells (SYCP3) (indicated with white arrows). Scale bars measure 25 μm. **(D)** Number of germ cells adhered to each organoid. Bars indicate mean ± SD, n = 3. Analysis was performed using one-way ANOVA followed by Tukey’s multiple comparison test. **(F)** Number of meiotic cells adhered to each organoid. Bars indicate mean ± SD, n = 3. Analysis was performed using unpaired two-tailed *t*-test. **(G)** Relative mRNA fold change of *Fshr*, *Shbg*, *Star*, *Hsd17b3* and *Kitlg* for organoids cultured at 34°C. Bars indicate mean ± SD, n = 3. Analysis for *Fshr*, *Star*, *Hsd17b3* and *Kitlg* was performed using one-way ANOVA followed by Tukey’s multiple comparison test and analysis for *Shbg* was performed using Kruskal-Wallis test with Dunn’s multiple comparison test. **(H)** Immunofluorescent detection of AR (indicated with white arrows) in the 34°C culture at day 0, day 14 and day 28. Scale bars measure 25 μm. *p* ≤ 0.05 (*), *p* ≤ 0.01 (**), *p* ≤ 0.001 (***), *p* ≤ 0.0001 (****). Only significant differences are indicated with asterisks.

Matsumura et al. (22) and Sato et al. (23) reported efficient spermatogenic differentiation of rodent testicular organ cultures at 34°C. To evaluate the effect of temperature on somatic cell and spermatogonial maturation, rat organoids were generated with OFM (72 hours: day 0 undifferentiated organoid) and then cultured with organoid differentiation medium (ODM) at 37°C and 34°C (22, 42) for up to 28 days. Immunofluorescence analysis revealed no morphological differences between the two groups at day 28 (**Figure 2A**) and both cultures showed expression of Claudin 11, a component of Sertoli cell tight junctions (**Figure 2B**) (43). Except for expression of *Shbg* (sex hormone binding globulin) (Sertoli and Leydig cells), which was increased 5.7-fold (n = 3, p < 0.05) in 34°C cultures, no significant differences in transcription levels were observed between the two groups for the somatic cell markers *Fshr* (follicle stimulating hormone receptor) (Sertoli cells), *Star* (steroidogenic acute regulatory protein), *Cyp17a1* (cytochrome P450 family 17 subfamily a member 1) (Leydig cells) and *Hsd17b3* (hydroxysteroid 17-beta dehydrogenase 3) (Sertoli and Leydig cells) (**Supplementary Figure 1A**) (n = 3, p > 0.05) (44–47). At day 7, rat testicular organoids contained UCHL1^+ve^ undifferentiated spermatogonia at both temperatures (**Supplementary Figure 1B**). Both at day 7 and 28, there was no difference in the number of VASA^+ve^ germ cells between 37°C and 34°C cultures (n = 3, p > 0.05) (**Figures 2C, D**). However, the number of VASA^+ve^ germ cells was lower at day 28 compared to day 7 at both temperature conditions (n = 3, p < 0.05) (**Figure 2D**). SYCP3^+ve^ spermatogenic cells were observed in both conditions, with a staining pattern similar to spermatocytes in 43-day old rat testes (**Supplementary Figure 1E**), starting from day 21. γ-H2AX, which is induced by the DNA double stranded break in leptotene and early zygotene, was also observed in both culture conditions with a staining pattern similar to 43-day old rat testes (**Supplementary Figures 1D, E**) (25). The number of SYCP3^+ve^ was quantified and their number was found to be higher at day 28 in the organoids cultured at 34°C compared to 37°C (n = 3, p < 0.05) (**Figures 2E, F**) (48, 49). Therefore, the 37°C cultures were excluded from further analysis.

To characterize maturation during the 28-day long culture, transcription levels of the immature Sertoli cell marker *Amh* (anti-mullerian hormone) and Sertoli and Leydig cell markers *Fshr*, *Shbg*, *Star*, *Hsd17b3* and *Kitlg* were analyzed for day 0, 7, 14, 21, 28 organoids. The transcription levels of *Amh* were undetectable within a week (n = 3, p < 0.05) while expression of *Cyp17a1* showed no significant changes over the duration of the culture (n = 3, p > 0.05). *Fshr*, *Shbg* and *Kitlg* were upregulated by 2.7-, 17.9- and 3.3-fold at day 21 (n = 3, p < 0.05) (**Figure 2G**). Transcription levels of *Star* and *Hsd17b3* increased 26.9- and 10-fold, respectively, by day 28 of culture (n = 3, p < 0.05) (**Figure 2G**). In addition, an increasing number of Sertoli cells in the organoids started to express AR with subsequent weeks of culture, indicating cell maturation (**Figure 2H**). Along with punctate SYCP3 staining (potentially leptotene spermatocytes) (**Figure 2E**), an elongated pattern of SYCP3 staining (as observed in early zygotene spermatocytes) (50) (**Supplementary Figure 1C**) was also detected at day 28. However, cells found with elongated SYCP3 expression were no longer adhered to the organoids at the time of analysis (**Supplementary Figure 1C**). In contrast, no PRM1^+ve^, TNP1^+ve^ or ACR^+ve^ cells were observed in the cultures.

### Rat Testicular Organoids to Model Reproductive Toxicity: Proof of Principle

**Figure 3:**
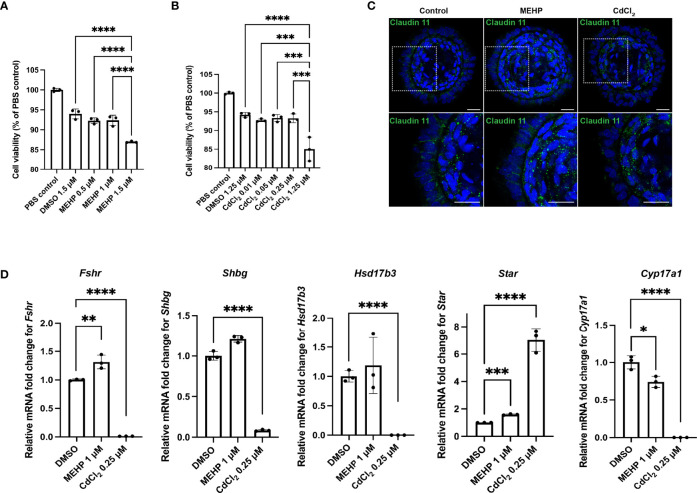
Rat testicular organoids allow for modeling reproductive toxicity. **(A, B)** Relative cell viability of organoids after MEHP and CdCl_2_ treatments. **(C)** Immunofluorescent images of day 21 rat testicular organoids treated with MEHP and CdCl_2_ showing tight junction protein (Claudin 11) (inserts on the top panels are showing the magnified area on the bottom panel). Scale bars measure 25 μm. **(D)** Relative mRNA fold change of *Fshr*, *Shbg*, *Star*, *Hsd17b3* and *Cyp17a1* in day 21 organoids after MEHP and CdCl_2_ treatment. Bars indicate mean ± SD, n = 3. Analysis was performed using one-way ANOVA followed by Tukey’s multiple comparison test. *p* ≤ 0.05 (*), *p* ≤ 0.01 (**), *p* ≤ 0.001 (***), *p* ≤ 0.0001 (****). Only significant differences are indicated with asterisks.

Initial dose-response experiments on monolayers of rat testicular cells (P4-P5) were used to select the dosages of 0.5, 1 and 1.5 μM for phthalic acid mono-2-ethylhexyl ester (MEHP); and 0.01, 0.05, 0.25 and 1.25 μM for the heavy metal cadmium chloride (CdCl_2_) to be tested on organoids. Then, relative cell viability assessments were performed on day 7 (treatment began on day 5) organoids treated with the aforementioned dosages with a 3-(4,5-dimethylthiazol-2-yl)-2,5-diphenyl-2H-tetrazolium bromide (MTT) assay, revealing that 1 μM MEHP and 0.25 μM CdCl_2_ were the highest doses without adverse effects on viability (n = 3, p < 0.05) (**Figures 3A, B**). Thus, all other dosages were excluded from further analyses. Since most of the maturation markers showed robust upregulation at day 21, toxicological effects of MEHP and CdCl_2_ were evaluated at day 21 by treating the organoids with MEHP and CdCl_2_ at day 19 and then harvesting and analyzing 48 hours later. MEHP treatment was associated with upregulation of *Fshr* and *Star* and downregulation of the expression of *Cyp17a1* (n = 3, p < 0.05) (**Figure 3D**). Expression of *Fshr*, *Shbg*, *Hsd17b3* and *Cyp17a1* was drastically downregulated upon exposure to CdCl_2_ (n = 3, p < 0.05) (**Figure 3D**). Transcription of *Star*, in contrast, was upregulated 7-fold compared to DMSO controls (n = 3, p < 0.05) (**Figure 3D**). In contrast to MEHP which led to partial loss of tight-junction protein Claudin11, CdCl_2_ treatment caused a total loss of Claudin 11 (**Figure 3C**).

## Discussion

In the last few years, we and others have reported the generation of testicular organoids from dissociated primary testicular cells (11, 14–18). In the current study, we adapted our previously established approach to generate porcine, murine, human and primate testicular organoids (11) to the formation of organotypic rat testicular organoids. Rats represent an important animal model for studying spermatogenesis and have been the main model for the study of reproductive toxicology (19–22). It was therefore necessary to establish the optimal conditions required for the generation and maturation of microwell-derived rat testicular organoids to support spermatogonial differentiation.

Rat testicular organoids presented some unique challenges that were not observed in our previously reported organoids (11). While porcine and murine organoids can be maintained for up to 45 days at ambient O_2_ tension, which translates to 18.4% or 6.34 mM O_2_ at 1084 m elevation in Calgary, Alberta, Canada (51, 52), rat organoids collapsed at day 6 by purging the interstitial compartment. This is likely due to the perturbation of the ratio of Sertoli cells to interstitial cells, caused by increased Sertoli cell proliferation. Sertoli cell proliferation levels are higher at high O_2_ tension compared to more hypoxic conditions (15%, 10%, 5% O_2_) (53). Unlike porcine and murine testicular organoids, where organoid formation leads to a contact inhibition effect on the cells, the Sertoli cells of rat testicular organoids seem to retain their ability to proliferate, which is exacerbated at ambient O_2_ tension. This increased number of Sertoli cells likely leads to a loss of their affinity for the basement membrane of the organoids. As a result, the Sertoli cells migrate to form separate aggregates which leads to complete or partial expulsion of the interstitial compartment.

Culture at 37°C can impair the glucose transport of spermatids and render spermatids and spermatozoa fragile (42, 54). It can also have deleterious effects on testis tissue *in vitro* (55). We observed higher expression of *Shbg*, increased numbers of early meiotic cells and the presence of cells with elongated SYCP3 staining pattern in 34°C culture conditions, indicating a positive effect on Sertoli cell maturation and spermatogonial differentiation, which is consistent with previous work (22, 45, 50, 54). As expected, number of germ cells were similar at both temperatures. This is consistent with previous reports which have found that the proliferation and survival of spermatogonia do not seem to be affected by temperature (54). However, the number of germ cells decreased over the duration of culture and zygotene spermatocytes, identified by the typical elongated staining pattern of SYCP3, were no longer adhered to the organoids. This gradual loss or dislodgement of loosely adhered germ cells is likely due to extensive media changes throughout the 28-day long culture. Such loss of a critical cell type may be mitigated by adapting the microwell system to support a continuous perfusion system (56). This would allow a slow and constant perfusion of media and reduce extensive handling for long-term cultures.

After establishing the optimal conditions for promoting maturation and early spermatogonial differentiation, we performed a proof of principle experiment to evaluate the utility of rat organoids for toxicological evaluation of drugs and environmental toxicants. Exposure to MEHP, a fairly common plasticizer (57), led to increased expression of *Fshr* and *Star* and decreased expression of *Cyp17a1*. This is consistent with previous reports, which have shown that phthalates can modulate basal steroidogenic machinery in both Sertoli and granulosa cells (58–60). Cadmium, a heavy metal that is often used as stabilizer in production of polymers and dyes, can cause endocrine disruption in the testis (61). We observed cadmium mediated disruption of Sertoli and Leydig cell function by downregulation of *Fshr*, *Shbg*, *Hsd17b3* and *Cyp17a1*, and upregulation of *Star*, which has been reported previously (61, 62). Both MEHP and CdCl_2_ are known to disrupt the blood testes barrier by downregulation of tight-junction proteins such as Claudin 11 (63, 64). We witnessed a similar effect upon exposure of organoids to MEHP and CdCl_2_. Human exposure to CdCl_2_ and MEHP can depend on a number of factors such as cumulative effects, metabolism by the liver, accumulation due to continuous environmental and occupational exposure (65, 66). While all of these considerations are beyond the scope of this current study, the dosages used here show definite disruptive effects on the steroidogenic machinery. This proof of principle experiment shows that the rat testicular organoids can serve as viable platforms for modeling male reproductive toxicity.

In conclusion, we report a rat testicular organoid system that reflect testis specific morphology and can support early testicular maturation. In addition, this system supports germ cell development to early meiosis up to the zygotene stage. Further optimization of the differentiation conditions may be warranted to support full *in vitro* spermatogenesis.

## Data Availability Statement

The original contributions presented in the study are included in the article/**Supplementary Materials**. Further inquiries can be directed to the corresponding author.

## Ethics Statement

The animal study was reviewed and approved by University of Calgary Animal Care Committee.

## Author Contributions

SS and ID conceived and designed the study. SS, NLML, and BH performed the data analysis, data interpretation and statistical analysis. SS prepared the manuscript. All authors contributed to the article and approved the submitted version.

## Funding

This work was supported by NIH/NICHD HD091068-01 to ID; a graduate student scholarship to SS from the Alberta Children’s Hospital Research Institute (ACHRI); and a Canadian Institutes of Health Research (CIHR) fellowship (MFE-176542) and an ACHRI postdoctoral fellowship to NLML.

## Conflict of Interest

The authors declare that the research was conducted in the absence of any commercial or financial relationships that could be construed as a potential conflict of interest.

## Publisher’s Note

All claims expressed in this article are solely those of the authors and do not necessarily represent those of their affiliated organizations, or those of the publisher, the editors and the reviewers. Any product that may be evaluated in this article, or claim that may be made by its manufacturer, is not guaranteed or endorsed by the publisher.
