# Supplementary material for: Organotypic Rat Testicular Organoids for the Study of Testicular Maturation and Toxicology
Source: Front Endocrinol (Lausanne). 2022 Jun 9;13:892342. doi: 10.3389/fendo.2022.892342 (PMC9218276; doi:10.3389/fendo.2022.892342)
Supplement: Supplementary file 1 [file DataSheet_1.docx]

Supplementary Material

# Supplementary Tables

**Supplementary table 1.** List of antibodies

| Antibody | Manufacturer | Catalog number | Lot number | Dilution |
| --- | --- | --- | --- | --- |
| GATA4 | Santa Cruz Biotechnology | sc25310 | E2215 | 1:200 |
| α-SMA | Abcam | ab7817 | GR309548-3 | 1:200 |
| 3β-HSD | Santa Cruz Biotechnology | sc30820 | J0516 | 1:100 |
| VASA | Abcam | ab27591 | GR3299257-3 | 1:100 |
| Collagen IV | Abcam | ab6586 | GR3215728-3 | 1:400 |
| Laminin | Abcam | ab11575 | GR233309-1 | 1:400 |
| Fibronectin | Abcam | ab23751 | GR273278-1 | 1:400 |
| Claudin 11 | Thermo Fisher | 36-4500 | RB215337 | 1:100 |
| AR | Abcam | ab74272 | GR99534-1 | 1:200 |
| SYCP3 | Santa Cruz Biotechnology | sc74569 | J1515 | 1:200 |
| TNP1 | Proteintech | 171781-1-AP | 00080847 | 1:200 |
| PRM1 | Proteintech | 15697-1-AP | 00006914 | 1:200 |
| ACR | Abcam | ab203289 | GR3209777-11 | 1:100 |
| γ-H2Ax | Cell Signaling | 2577S | 11 | 1:400 |
| UCHL1 | Abcam | ab8189 | GR3351033-3 | 1:100 |
| Donkey anti-Rabbit IgG (H+L) Highly Cross-Adsorbed Secondary Antibody, Alexa Fluor 488 | Thermo Fisher Scientific | A21206 | 1754421 | 1:500 |
| Donkey anti-Mouse IgG (H+L) Highly Cross-Adsorbed Secondary Antibody, Alexa Fluor 555 | Thermo Fisher Scientific | A31570 | 1048568 | 1:500 |
| Donkey anti-Goat IgG (H+L) Cross-Adsorbed Secondary Antibody, Alexa Fluor 488 | Thermo Fisher Scientific | A11055 | 1827671 | 1:500 |

**Supplementary table 2.** List of primers

| Target gene | Direction | Sequence (5’-3’) |
| --- | --- | --- |
| *Gapdh* | Forward | TCACCACCATGGAGAAGGC |
|  | Reverse | GCTAAGCAGTTGGTGGTGCA |
| *Amh* | Forward | CAACCAAGCAAAGAAGGTGCC |
|  | Reverse | CAGCGGGAATCAGAGCCAAA |
| *Fshr* | Forward | CCTCTGGGCCAGTCATTTTAGAT |
|  | Reverse | GCCTCCATGAGGGTGACAAA |
| *Shbg* | Forward | GTTGCCGACTGCTTCTGTTGT |
|  | Reverse | CTCAAAGGAGGAAGAGGGTTTGCT |
| *Star* | Forward | GCGGAACATGAAAGGACTGAG |
|  | Reverse | TGTAGGACAGCTCCTGGTCA |
| *Hsd17b3* | Forward | GATGGCATCGGGAAAGCCTA |
|  | Reverse | GCTTCCAGTGGTCCTCTCAAT |
| *Cyp17a1* | Forward | CCAGGGAGGTGCTCATCAAG |
|  | Reverse | CAAGAGGCTTTGAGTCACCATC |
| *Kitlg* | Forward | TATGTTACCCCCTGTTGCAG |
|  | Reverse | GGACTTTGCGGCTTTCCTATTA |

# Supplementary Figures


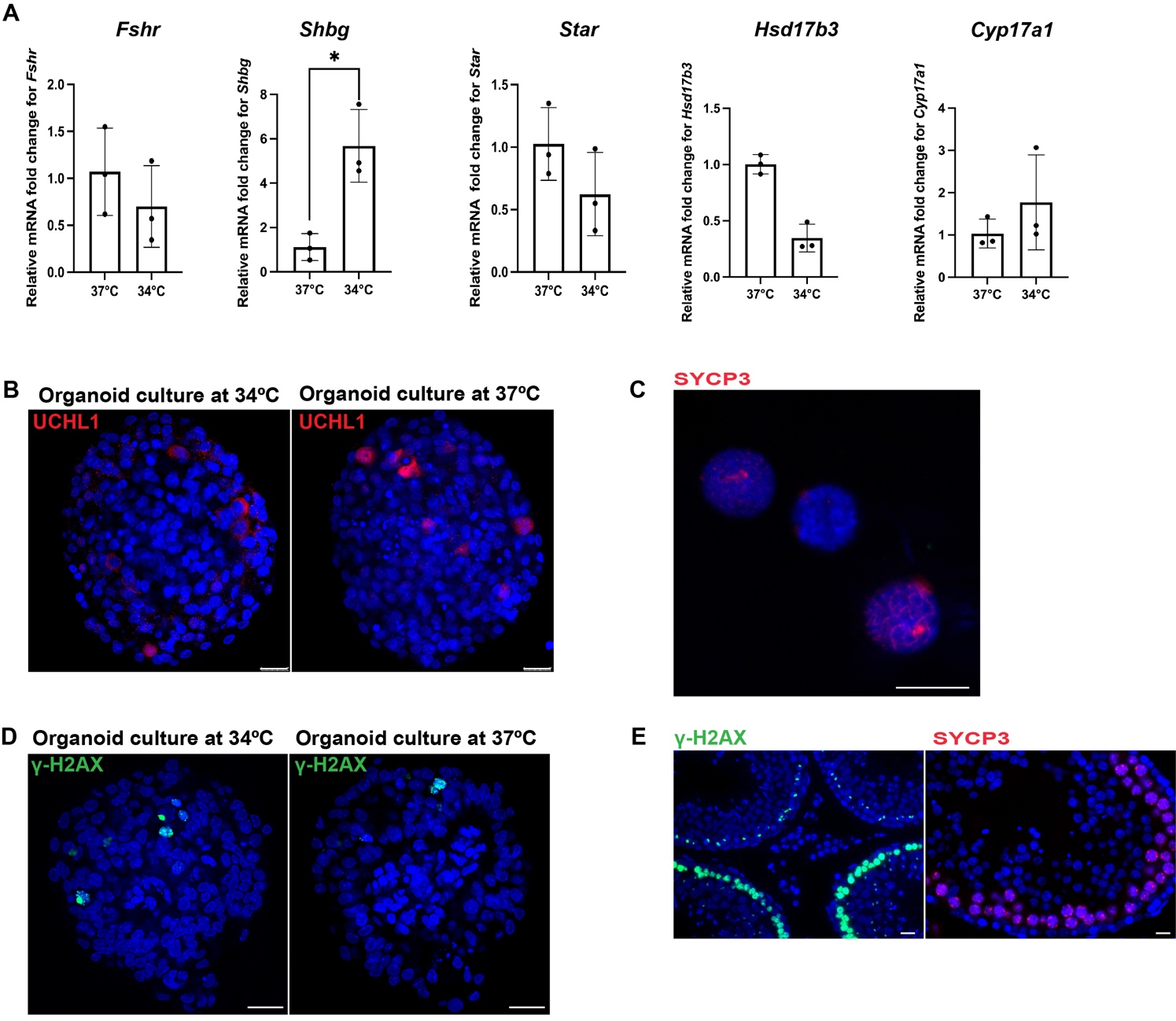


**Supplementary Figure 1.** **(A)** Relative mRNA fold change of *Fshr*, *Shbg*, *Star*, *Hsd17b3* and *Cyp17a1* for organoids cultured at 37°C and 34°C. Bars indicate mean ± SD, n = 3. Analysis was performed using unpaired two-tailed *t*-test. *p* ≤ 0.05 (*). Only significant differences are indicated with asterisks. **(B)** Immunofluorescent image of UCHL1 (undifferentiated spermatogonia) in 34°C and 37°C cultures. Scale bar measures 25 μm. **(C)** Immunofluorescent image of elongated SYCP3^+ve^ staining pattern in 34°C culture. Scale bar measures 10 μm. **(D)** Immunofluorescent image of γ-H2AX in 34°C and 37°C cultures. Scale bar measures 25 μm. Scale bars measure 25 μm. **(E)** Immunohistochemistry for γ-H2AX and SYCP3 in 43-day old rat testes. Scale bars measure 25 μm.
